# Improving outcomes in adult patients who self-harm—evaluating a brief psychological intervention in emergency departments (ASSURED): protocol of a randomised controlled clinical trial

**DOI:** 10.1186/s13063-025-09411-7

**Published:** 2026-01-30

**Authors:** Rose McCabe, Mimi Suzuki, Sally O’Keeffe, Neil Walker, Richard Hooper, Stefan Priebe, Borislava Mihaylova, Yan Feng, Chris Dickens, Peter Aitken, Vera Araújo Soares, Richard Byng, Domenico Giacco, Navneet Kapur, Will Lee, Peter Riou, Mary Ryan, Alan Simpson, Helen Smith, Lucy Polyanna Goldsmith, Alexandra Elissavet Bakou

**Affiliations:** 1 School of Health and Psychological Sciences, City St George’s, University of London, London, UK; 2 Faculty of Medical Sciences, Newcastle University, Newcastle upon Tyne, UK; 3 Pragmatic Clinical Trials Unit, Queen Mary University of London, London, UK; 4 Health Economics and Policy Research Unit, Queen Mary University of London, London, UK; 5 University of Exeter, Exeter, UK; 6 Division of Prevention, Center for Preventive Medicine and Digital Health (CPD), Medical Faculty Mannheim, Heidelberg University, Heidelberg, Germany; 7 University of Plymouth, Plymouth, UK; 8 University of Warwick, Warwick, UK; 9 University of Manchester, Manchester, UK; 10 Royal Devon University Healthcare NHS Foundation Trust, Exeter, UK; 11 King’s College London, London, UK; 12 University Hospitals Plymouth NHS Trust, Plymouth, UK; 13 Mental Health Policy Research Unit, University College London, London, UK

**Keywords:** Self-harm, Non-suicidal self-injury, Suicidal ideation, Emergency department, Narrative interviewing, Safety plan, Psychological intervention, Solution-focused brief therapy, Randomised controlled clinical trial, Protocol

## Abstract

**Background:**

Patients with self-harm and suicidal ideation are increasingly presenting in emergency departments (ED) in the UK. Self-harm is the strongest risk factor for suicide. Currently, there are no evidence-based interventions for self-harm offered in the context of general hospitals in the UK. This trial, funded by the National Institute for Health and Care Excellence (NIHR), aims to assess the clinical and cost-effectiveness of the ASSURED intervention. The ASSURED intervention includes up to five rapid follow-up contacts, comprising a narrative interview and enhanced safety planning session and three solution-focused sessions. The trial is sponsored by Devon Partnership NHS Trust and City St George’s, University of London.

**Methods:**

ASSURED is a multicentre, two-arm, parallel-group, individually randomised, controlled trial comparing the ASSURED intervention with usual care. The primary outcome is whether study participants re-attend ED and are referred to liaison psychiatry within 18 months from the date of randomisation. Secondary outcomes include suicidality, self-reported self-harm, psychological wellbeing, social outcomes, experiences of attending the ED, and suicide. The study will also evaluate the cost-effectiveness of the intervention. The aim of this study was to recruit and randomise 620 patients across 14 acute hospital sites in London, Devon, Somerset, and the Midlands. Participants are invited to complete research assessments at baseline and 3, 9, and 18 months. The first participant was enrolled in the study in August 2022, and the recruitment target was met in December 2024.

**Discussion:**

This will be the first UK trial to test the effectiveness and cost-effectiveness of a rapid intervention for patients presenting to EDs with self-harm and suicidal ideation and has the potential to improve outcomes for these patients.

**Trial registration:**

ISRCTN 13472559. Registered on 18 of November 2021.

**Supplementary Information:**

The online version contains supplementary material available at 10.1186/s13063-025-09411-7.

## Introduction

### Background and rationale {9a}

Death by suicide is a catastrophic loss of life for the person and the people they leave behind: children, partners, family, and friends. Self-harm and suicidal ideation are key risk factors for suicide. Self-harm refers to intentional self-poisoning or self-injury, irrespective of the apparent purpose of the act [1]. For patients, self-harm is associated with significant personal suffering, poor mental health, poor quality of life, and needs for support from carers and services [2, 3]. Each year, approximately 220,000 episodes of self-harm by 150,000 people are managed by emergency departments (EDs) in England [4]. The Government’s Suicide Prevention Strategy for England has prioritised support for people who self-harm and highlighted the role of EDs [5]. In addition to the high numbers of people presenting to EDs with self-harm, there are increasing numbers of people presenting to EDs with suicidal ideation, which is also an important risk factor for suicide [6, 7]. Both those presenting to the ED with self-harm and suicidal ideation have been identified as warranting personalised management plans and active follow-up to reduce the risk of self-harm [6, 8].

In England’s National Health Service (NHS), the National Institute for Health and Care Excellence (NICE) recommends a psychosocial assessment by specialist mental health practitioners in the ED for people who present with self-harm [9]. Most EDs have a psychiatric liaison team staffed by specialist mental health practitioners. They conduct psychosocial assessments to support patients, assess their current and future health and social care needs, and make onward referrals. While many people need further support, accessing specialised mental health services is often not a realistic option because (a) there is not enough capacity in these services, (b) specialised treatment is costly, and (c) many patients do not attend or withdraw from treatment [10]. Research has found that patients often feel stigmatised when seeking help in the ED for self-harm and suicidal ideation [11] and often feel care plans are not personalised or meaningful to them [12]. Furthermore, there are substantial gaps in care provision for these patients, who are often discharged without a new or active referral to mental health services [13].

The present study is part of a research programme called ASSURED which seeks to address this gap in care provision. The overall aim of the ASSURED study is to develop and trial a rapid and cost-effective intervention to improve outcomes for patients presenting to the ED with self-harm and/or suicidal ideation. The ASSURED intervention was developed based on international evidence [14], focus groups, and semi-structured interviews with stakeholders and ongoing input from a lived experience advisory group [11]. The intervention was designed to consist of an enhanced psychosocial assessment in the ED (comprising a narrative interview and enhanced safety plan), three solution-focused follow-up sessions over 8 weeks, a fifth optional session, and three personalised letters sent out over 9 months post-randomisation.

The intervention was tested in a feasibility study and followed by an internal pilot trial, conducted from July 2022 to March 2023 across four NHS hospitals in England with patients presenting with self-harm and/or suicide [15]. The original study was a cluster randomised controlled trial with participating practitioners allocated to ASSURED or treatment as usual (TAU) and the first intervention session taking place in the mental health psychosocial assessment in the ED. However, the Covid-19 pandemic meant ED practitioners’ workload increased significantly, and researchers had no access to ED sites to gain consent from patients before their psychosocial assessment. Hence, the pre-COVID cluster randomised controlled trial had to be modified. The key changes were moving to individual patient randomisation, patient consent soon after ED attendance, and delivering the first intervention session approximately 2 weeks after ED attendance. This removed reliance on ED and liaison staff to screen/consent patients.

### Objectives {10}

The objectives of the ASSURED trial are to (A) assess whether the intervention reduces the need for mental health reattendance at the emergency department; (B) assess whether the intervention improves secondary outcomes: mental health outcomes are suicidality (suicidal thoughts and/or plans), self-reported self-harm, psychological wellbeing, and suicide (death due to self-inflicted self-injury or self-poisoning) [16], social outcomes (accommodation, employment, partnership/family, friendships), and experiences of attending the ED; and (C) assess the costs and cost-effectiveness of the intervention.

### Patient and public involvement {11}

Patient and public involvement (PPI) has been a crucial aspect of all aspects of the study. A person with lived experience is a co-applicant on the project and part of the Programme Management Group (PMG). A Lived Experience Advisory Panel (LEAP) was set up to inform the ASSURED project, comprising a diverse group of seven people with lived experience of self-harm and/or suicidality. The group has been enthusiastic to be involved in all aspects of the project. Frequency of meetings is flexible depending on the phase of the study.

The LEAP has contributed to all aspects of the project—from designing the study logo, co-producing intervention and training materials (including a safety plan and consent forms), contributing to training practitioners, and advising on different aspects of trial procedures and dissemination. Training to participate in data analysis will be offered, with ongoing supervision and guidance from researchers during the analysis process. We will request permission from the ethics committee to consider LEAP members trained in data analysis and confidentiality as members of the research team. We would request permission for LEAP members to analyse transcripts of anonymised focus groups/interviews.

LEAP meetings are facilitated by a qualified, experienced mental health practitioner and a person with lived experience, who support a culture of open reflective communication and trust, which is modelled by the facilitators themselves. The LEAP members work together very openly, encouragingly, and cooperatively, valuing one another and making valuable contributions to the research. Open communication about any concerns or upsetting experiences or emotions is actively encouraged, with staff and facilities available to provide emotional support.

### Trial design {12}

ASSURED is a multicentre, two-arm, parallel-group, individually randomised controlled trial comparing the ASSURED intervention with usual care. Patients are randomised 1:1 in random block sizes of 4, 6, or 8 using stratification by site and type of presentation, i.e. self-harm or suicidal ideation.

## Methods: participants, interventions, and outcomes

### Study setting {13}

Recruitment for the trial takes place after patients have been seen by the liaison psychiatry teams for the standard psychosocial assessment in 14 EDs in London (Homerton University Hospital, Royal London Hospital, Newham University Hospital, Whipps Cross Hospital, University College Hospital, North Middlesex University Hospital), Surrey (East Surrey Hospital), Devon (Royal Devon and Exeter Hospital, Torbay Hospital), Somerset (Musgrove Park Hospital, Yeovil District Hospital), and the Midlands (University Hospital Coventry and Warwickshire, George Eliot Hospital, Warwick Hospital). These emergency department sites are covered by 10 different liaison psychiatry teams across East London NHS Foundation Trust, Surrey and Borders NHS Trust, Devon Partnership NHS Trust, Coventry and Warwickshire Partnership NHS Trust, North East London NHS Foundation Trust, Camden and Islington NHS Foundation Trust, Barnet, Enfield and Haringey Mental Health Trust, and Somerset NHS Foundation Trust. These hospital sites were approached based on existing annual estimates of self-harm presentations in the ED as well as the capacity and resources within the liaison psychiatry teams to undertake the study.

### Eligibility criteria: eligibility criteria for participants {14a}

#### Patient inclusion criteria

1.  ≥ 18 years of age
2. Presenting to the ED and referred to the liaison psychiatry team for a psychosocial assessment
3. Presenting with self-harm, i.e. an intentional act of self-poisoning or self-injury, irrespective of the motivation or apparent purpose of the act *and/or* presenting with suicidal ideation, i.e. thoughts of not wanting to live
4. Provide informed consent to take part

#### Patient exclusion criteria

1. Experiencing a psychotic episode
2. No capacity to provide written informed consent
3. Needing an interpreter, due to the additional resource required
4. Ministry of Justice patients subject to a restriction order
5. Living out of trust (to ensure access to medical records)
6. Safety concerns

### Eligibility criteria: if applicable, eligibility criteria for sites and for individuals who will deliver the interventions (e.g. surgeons and physiotherapists) {14b}

The ASSURED intervention is delivered by NHS practitioners working or allied with liaison psychiatry teams (e.g. mental health practitioners, psychiatrists, doctors, support workers, psychologists, nurses, occupational therapists) as well as experienced assistant psychologists and research assistants working or affiliated (i.e. holding honorary contracts) within the participating NHS teams.

### Intervention and comparator: intervention and comparator with sufficient details to allow replication including how, when, and by whom they will be administered {15a}

Participants in the control group receive usual care. This may involve discharge to the general practitioner, referral or discharge to secondary mental health care, or psychiatric admission. Given the modified study design, it is possible that participants randomised to the control group may have received the standard psychosocial assessment by a practitioner trained in the ASSURED intervention. It is unlikely that these practitioners will deliver the additional ASSURED components (narrative interview, enhanced safety planning) due to time constraints and discharge targets in the ED. In the unlikely event that ASSURED components may be implemented in the control arm in the psychosocial assessment, any impact should be mitigated by randomisation.

Practitioners are trained in the ASSURED intervention. The ASSURED intervention manual is published online (https://assuredstudy.co.uk/manual/). In addition to usual care, the intervention involves rapid follow-up and up to five follow-up sessions over 8 weeks. Sessions are ~50 min long and are delivered flexibly depending on the person’s preference (face to face at the hospital site if a room is available, over the phone, or by video call) with (as far as possible) the same practitioner around 1 week, 2 weeks, 4 weeks, and 8 weeks after randomisation with a fifth optional bank session. These timescales are a guideline and may be used flexibly depending on the availability of the patient and practitioner.

In ASSURED, the use of language and active listening is very important. The training emphasises the importance of human connection, elaboration, validation of distress and help-seeking, and giving hope. Practitioners are trained to stay close to the person’s own words, ask open questions, and wait for people to respond and validate their difficult experiences. Practitioners are trained to use these skills in the therapeutic sessions.

In the first session, the practitioner uses narrative interview techniques to explore the person’s story [17]. The practitioner and patient then develop a personalised and enhanced safety plan [18] in the patient’s own words. The aim of the safety plan is to identify warning signs and consider steps that the person can take to manage distressing experiences such as having suicidal thoughts. These are described under “Distractions” (what can I do to distract myself?), “Changing my Environment” (where can I go to distract myself from thoughts?), “People I trust” (“Who can I contact when I feel overwhelmed?”), and “Professionals” (“Which professionals can I contact?”). The session is supported by a template that participants and practitioners complete together either online or on paper.

The patient is then offered three further solution-focused sessions [19] to explore the patient’s future hopes, along with resources and strengths already present in achieving or working towards their future hopes. The participant is asked on a scale from 0 to 10 where they are in terms of their best hopes (“If 10 is your best hopes are realised, and 0 is the opposite, where are you now on the scale?”) and how confident they feel that they can achieve their best hopes (“If 10 is – you are as confident as you can be in making progress towards your best hopes – and 0 is the opposite, where is your confidence now on the scale?”). A list of signposting resources has also been developed as part of the ASSURED intervention, and practitioners are encouraged to discuss further support with participants and, where relevant, spend 5 min at the beginning of every session checking in on any referrals made to other services. Within the ethos of the solution-focused approach, if a person misses a session, this is not perceived as nonengagement or patient “resistance”. Practitioners record the missed session, attempt to contact the patient, and reschedule the session as flexibly as possible.

The optional bank session can occur up to 9 months post-randomisation, following the solution-focused approach. The patient receives three personalised letters from the practitioner ending at 9 months to let the patient know that the practitioner is thinking of them and to remind them of the safety plan, bank session, and support networks.

### Intervention and comparator: criteria for discontinuing or modifying allocated intervention/comparator for a trial participant {15b}

There may be instances where a clinical decision is made for the patient to discontinue their involvement in the study, e.g. services may deem it likely that new mental health practitioners being introduced to a participant’s existing care could be destabilising. It is expected that this will disproportionately affect intervention participants given that there will be more contacts with the study clinicians compared to control participants. If a practitioner or a researcher deems that the patient’s risks have escalated during their participation in the study, then this will be raised immediately with the local principal investigator (PI). A decision will be made as to whether it is appropriate for the patient to continue with the intervention and research assessments. Patients enrolled in the study will still be followed up for the primary outcome (ED reattendance with referral to liaison psychiatry) unless they explicitly request that researchers do not extract their data from the medical records.

### Intervention and comparator: strategies to improve adherence to intervention/comparator protocols, if applicable, and any procedures for monitoring adherence {15c}

To improve patient adherence to the intervention, practitioners will aim for flexibility in the time and delivery of sessions. Attendance at sessions will be monitored using a form completed by practitioners. Practitioners receive 2 days of training in the intervention, conduct role-plays with trainers, and attend regular supervision. The training takes place online, usually in groups with other participating practitioners, and it is facilitated by an experienced study researcher (chief investigator, trial manager, senior research assistant). Two drop-in, peer-supervised sessions are offered every week. To mitigate against contamination, participating practitioners are reminded of the importance of not sharing details of the ASSURED intervention with other colleagues.

In addition, study researchers routinely collect data on the training and supervision that practitioners receive, i.e. date of training/supervision, length of training/supervision (mins), type of training (core, top-up, one-off), mode of training/supervision (face to face, videocall with facilitation, telephone, email, website self-learning, and other), and travel required. Data is routinely collected for all study participants in the intervention arm on intervention sessions, i.e. practitioner delivering the intervention, number of intervention sessions delivered, date of intervention session, mode (face to face, video call, telephone call), and length (mins). Fidelity to the intervention will be assessed via video and audio recordings of the intervention session where participants have provided informed consent.

### Intervention and comparator: concomitant care that is permitted or prohibited during the trial {15d}

Trial participants are permitted to receive any concomitant care during the trial.

### Outcomes {16}

The primary outcome is a binary outcome [yes (y = 1) or not (y = 0)], i.e. whether study participants re-attend ED and are referred to liaison psychiatry within 18 months from the date of randomisation. Re-attendance with referral to liaison psychiatry is any (a) attendance for self-harm or other mental health crisis, (b) a referral is made and accepted by the liaison team, and (c) the attendance and referral happen in the same ED visit. This data (i.e. date and time of referral to liaison psychiatry after ED attendance) will be obtained from NHS business intelligence teams and may be supplemented/cross-referenced with NHS England. A pre-specified data extraction protocol has been agreed upon with the NHS Business intelligence teams.

The advantage in using this method of data extraction is that the data will be available for almost all participating patients and independent of rater bias. This outcome is linked to Objective A (see the “Objectives{10}” section) and reflects the severity and impact of repeat self-harm and is widely available from routine service data. The extraction of the primary outcome data through the NHS business intelligence teams was partially piloted in the internal pilot phase of the trial [15] and is being validated across participating sites to ensure reliability in the data extraction process.

In addition to the secondary outcomes described below, during the baseline assessment, we collect sociodemographic information for participants on gender, country of birth, ethnic group, physical health conditions, psychiatric diagnosis, previous contact with mental health services, psychiatric hospital admissions, level of education, marital status, type of accommodation, living situation, dependents, employment status, receipt of state benefits, treatment history (including contact with healthcare professionals and general practitioners for self-harm and contact with mental health services), asylum-seeker status, and drug- and alcohol-related problems.

The following secondary outcomes will be assessed:

1. Total number of patient attendances to the emergency department over 18 months following randomisation, extracted from NHS England. This outcome is linked to Objective C (see the “Objectives {10}” section).
2. Self-reported self-harm, using text messages at approximately 1, 2, 3, 9, and 18 months. Participants who have consented to this option are approached via text messages and invited to answer the following questions: “Have you self-harmed in the past month?” (yes/no) and “How many times?” (numerical response). This outcome is linked to Objective B (see the “Objectives {10}” section).
3. Suicidal ideation, using the Beck Scale for Suicide Ideation (BSSI) [20] at 3, 9, and 18 months [20, 21]. This is a 21-item scale evaluating the presence and intensity of suicidal intent in the week before the evaluation. This scale shows good concurrent validity and reliability, with a Cronbach’s alpha value of *α* > 0.90. This outcome is linked to Objective B (see the “Objectives {10}” section).
4. Use of primary and community care, medications, social services, productivity loss, and family costs will be collected using the Client Service Receipt Inventory (CSRI), administered at baseline, 3, 9, and 18 months. These outcomes are linked to Objective C (see the “Objectives {10}” section).
5. Psychological distress measured with the Clinical Outcomes in Routine Evaluation-Outcome Measure (CORE-OM) [22, 23] at 3, 9, and 18 months. Coefficient *α* for all domains is > 0.75 and < 0.95 [24]. This outcome is linked to Objective B (see the “Objectives {10}” section), and CORE-OM was selected to capture global distress beyond suicidality.
6. Quality of life measured with EuroQol-5 Dimension-5 Level tool (EQ-5D-5L) [25] at 3, 9, and 18 months. This tool shows established test–retest reliability and convergent validity [23, 24]; in a systematic review of the EQ-5D-5L psychometric properties, eight studies have reported excellent scale agreement (*ICC* > 0.075). A great number of papers have reported strong, along with expected, correlations with other measures of health; the strongest correlations have been observed for multi-attribute utility instruments (*pooled rho* = 0.756), physical/functional measures (*pooled rho* = 0.582), and pain/discomfort measures (*pooled rho* = 0.595). This outcome is linked to Objective B (see the “Objectives {10}” section).
7. Social outcomes, measured with the Social Outcomes Index (SIX) [26] at 3, 9, and 18 months. This 4-item tool measures social outcomes in four domains: employment (none, 0; voluntary/protected/sheltered work, 1; regular employment, 2), accommodation (homeless or 24 h supervised, 0; sheltered or supported accommodation, 1; independent accommodation, 2), partnership/family (living alone, 0; living with a partner or family, 1), and friendship (not meeting a friend within the last week, 0; meeting at least one friend in the last week, 1). The resulting score of SIX ranges from 0 to 6. SIX was selected to capture the person’s social situation (accommodation, employment, family/partnership, friendships), which can be affected by a mental health crisis. This outcome is linked to Objective B (see the “Objectives {10}” section).
8. Psychological wellbeing—Short Warwick–Edinburgh Mental Wellbeing Scales (SWEMWBS) at 3, 9, and 18 months [27]. This is a 7-item scale measuring feeling and functioning changes. SWEMWBS captures positive aspects of mental health, such as optimism, relaxation, and social connectedness, which may be increased by solution-focused approaches. Cronbach’s alpha of > 0.70 indicates acceptable internal reliability [28]. This outcome is linked to Objective B (see the “Objectives {10}” section).
9. Experiences of care in Accident and Emergency Questionnaire, devised for this study at the baseline research assessment soon after ED attendance. This outcome is linked to Objective B (see the “Objectives {10}” section).
10. Death by suicide, i.e. the cause of death is intentional self-harm or undetermined intent derived from NHS/local authority/coroner records at 18 months. This outcome is linked to Objective B (see the “Objectives {10}” section).
11. All-cause mortality at 18 months after randomisation, extracted from NHS England Data. This outcome is linked to Objective B (see the “Objectives {10}”section).

The mediator for the primary outcome is the therapeutic relationship, which is self-rated by patients on the Helping Alliance Scale [29], which has been adapted for the ED context. This scale is completed at 3 months and by the participants in the intervention group only.<div class="NodiCopyInline">The mediator for the primary outcome is the therapeutic relationship, which is self-rated by patients on the Helping Alliance Scale [29], which has been adapted for the ED context. This scale is completed at 3 months and by the participants in the intervention group only.</div>

### Harms {17}

We record adverse events and serious adverse events to ensure that any possible harms are detected and monitored appropriately. Reporting of events starts from the point at which the participant is enrolled in the study (i.e. both the consent and baseline procedures have been completed) until the end of their involvement in the study (i.e. the date of the last participant data collection). Adverse and serious adverse events may be detected either via data collection procedures (i.e. researcher assessments and interviews taking place in person or via phone/video call) or via incidental reporting (i.e. researchers may become aware that a trial participant has re-attended the ED while they are on site or be alerted to a potential adverse event by a member of staff in the ED or a participating practitioner). 

The chief investigator (CI) will ensure that safety monitoring and reporting are conducted in accordance with the study procedures. Adverse events and serious adverse events are recorded on a case report form and reviewed by the principal investigator (PI) to determine the relatedness, expectedness, and severity of the events. Any member of staff who becomes aware of an adverse and serious adverse event as described in the protocol must report it to the PIs immediately or the CI and/or sponsor Representative if the PI is not available, with as much information that is available at the time. A research team member can be delegated the task of establishing resolution of the event and completing the protocol-specific adverse event/serious adverse event reporting log. In the event where researchers are unsure of whether an event is considered expected/procedural, they will notify the senior research assistants or trial manager and seek advice.

Summaries of adverse events are periodically (and at least twice a year) presented to the study sponsor, the PMG, the DMC, and the PSC. Serious adverse events which are definitely, probably, or possibly related to participation in the trial and unexpected (RUSAE) are reported to the Research Ethics Committee and the study sponsor within 15 days of the CI becoming aware of the event.

### Participant timeline {18}

A timeline for ASSURED is shown in Table 1.

**Table 1:** Schedule of enrollment, interventions, and assessments

	Trial period								
	Enrollment			Post-randomisation (months)					
Timepoint	Pre-randomisation	0	1	2	3	6	9	18	> 18 months post-randomisation
**Enrollment**									
Eligibility screening from liaison psychiatry records	X								
Confirmation of eligibility and informed consent	X Approximately 2 weeks of ED attendance								
Randomisation		X							
**Intervention/comparator**									
**ASSURED intervention** (narrative interviewing & enhanced safety planning session, three solution-focused sessions over 8 weeks, three follow-up letters over 9 months, optional bank session)		X	X	X	X	X	X		
**Treatment as usual**		X	X	X	X	X	X	X	
**Assessments**									
**Research assessment at baseline** (sociodemographic information—gender, country of birth, and ethnic group—physical health conditions, psychiatric diagnosis, previous contact with mental health services, psychiatric hospital admissions, level of education, marital status, type of accommodation, living situation, dependents, employment status, receipt of state benefits, treatment history (including contact with healthcare professionals and general practitioners for self-harm and contact with mental health services), asylum-seeker status, drug- and alcohol-related problems, CORE-OM, BSSI, CSRI, SIX, SWEMWBS, EQ-5D-5L, and experiences of care in accident & emergency)	X								
**Research assessment at 3 months** CORE-OM, BSSI, CSRI, SIX, SWEMWBS, EQ-5D-5L and for participants in the intervention arm, the Helping Alliance Questionnaire					X				
**Research assessment at 9 months** CORE-OM, BSSI, CSRI, SIX, SWEMWBS, EQ-5D-5L							X		
**Research assessment at 18 months** CORE-OM, BSSI, CSRI, SIX, SWEMWBS, EQ-5D-5L								X	
Self-reported self-harm over the past month Via text messages			X	X	X		X	X	
Intervention contacts (including duration and mode of contact)			X	X	X	X	X	X	
Repeat re-attendance to ED with a referral to liaison psychiatry (primary outcome—binary outcome) via NHS Trust Data Download & NHS England									X
All patient attendances to ED for 18 months pre- and post-randomisation via NHS England	X								X
Health care use via NHS England									X
All-cause mortality (including suicide) via NHS England									X

### Sample size {19}

Around 30% of patients attending the ED with self-harm/suicidal ideation re-attend within 18 months. Reattendance rates are similar for self-harm and suicidal ideation presentations [6]. Reducing this by one-third (i.e. from 30% to 20%) would be a clinically important difference.

The original sample size calculation for the trial was based on a cluster-randomised design with practitioners as clusters. A figure of 0.03 for the intracluster correlation (ICC) was assumed based on published literature [30]. This gave a target sample size of 1088 to achieve 90% power at the 5% significance level, assuming 12 patients in each cluster. When the protocol was amended to individual patient randomisation, we also considered clustering in the intervention arm since practitioners are directly involved in delivering the intervention. However, practitioner-level clustering in a trial of a similar intervention with the same primary outcome was negligible [31, 32], suggesting the original figure of 0.03 for the ICC was an overestimate. The revised calculation (conducted blind to any interim results from the trial) assumed a value of 0.01. With the agreement of the PSC, the power was also reduced from 90% to 80% for the revised calculation, giving a target sample size of 620 (310 in each arm). Due to the impact of COVID-19 (i.e. significantly increased pressure on EDs and diverting mental health patients from EDs), this was a pragmatic decision to address difficulties in recruitment given the challenges of conducting research in addition to clinical care in the ED. The calculation was performed using the “clsampsi” package in Stata (Stata Corporation, College Station, TX, USA) [33]. 

### Recruitment {20}

We aim to randomise 620 adult patients who present to the emergency department over 27 months. Patients were invited to participate in the study either at the point of attending the emergency department by a practitioner or shortly after leaving the emergency department by a local NHS researcher. We aim to enrol and randomise patients within 2 weeks of having attended the ED.

## Methods: assignment of interventions

### Randomisation

#### Sequence generation, allocation concealment mechanism, and implementation {21 a, b, 22, 23}

Randomisation is implemented using the randomisation module of the REDCap electronic data capture tools hosted at Queen Mary University of London to ensure allocation concealment. The allocation sequence is generated by an independent statistician at the Pragmatic Clinical Trials Unit, using STATA. Allocation to intervention or control is in a 1:1 ratio and in random block sizes of 4, 6, or 8, stratified by site and type of presentation (self-harm or suicidal ideation). The central team access the REDCap module to determine allocations, which they communicate to researchers at the respective sites who then inform patients.

#### Blinding {24 a, b, and c}

The statisticians and health economists analysing the trial data will remain blind to patient allocation until the Statistical Analysis Plan and Health Economics Analysis Plan have been signed off and the trial database finalised and locked for analysis. The researcher who recruits each patient will be unblinded to the patient’s allocation at the point of randomisation (which is after the baseline assessment). We do not anticipate blinding being an issue for the primary outcome as ED reattendances will be extracted from medical records. Similarly, this should not be an issue for the secondary outcome measures, as the majority are based on direct patient self-report. Participants are not blinded.

## Data collection, management, and analysis

### Data collection methods: plans for assessment and collection of outcomes {25a}

All potentially eligible patients were approached for consent to join the study after ED attendance. The baseline research assessment took place after consent and prior to randomisation. Participants will be asked to complete further assessments at 3-, 9-, and 18-month post-randomisation. A subsample of approximately 30 patients, up to 30 practitioners, approximately 10 carers or trusted others (if available), and up to 10 team managers and local PIs such as liaison psychiatry team managers will be approached for a qualitative interview to explore experiences of receiving/delivering the intervention. Further details about the interviews are presented under section “Process evaluation—qualitative interviews”.

### Data collection methods: plans to promote participant retention and complete follow-up {25b}

Follow-up assessments at 3, 9, and 18 months will be conducted by researchers at City St George’s, University of London, Warwick University, and Devon Partnership NHS Trust. The study has a dedicated website (https://assuredstudy.co.uk/) and study X feed (@ASsuREDproject) to facilitate engagement with the study.

### Data management {26}

The participant ID will be used on all case report forms (CRFs) for both patient and practitioner participants. Researchers at City St George’s, University of London, Warwick University, and Devon Partnership NHS Trust will enter data into an electronic database. For each researcher, the study team will review the first five case report forms (CRFs) they enter into the database to verify accuracy.

### Statistical methods: primary and secondary outcomes {27a and b}

All analyses will adhere to the intention-to-treat principle unless otherwise stated. The primary outcome (i.e. ED reattendance with a referral to liaison psychiatry within 18 months [a binary outcome—yes or no]) will be analysed using a mixed-effects logistic regression model, with random intercepts for (i) hospital sites (this refers to the different liaison psychiatry teams) and (ii) clinicians. The model will be adjusted for the following factors/covariates: hospital site, a binary indicator of whether the participant presented with suicidal ideation only or evidence of self-harm at baseline, and number of hospital attendances for self-harm/suicidal ideation over the 18 months prior to baseline. Secondary outcomes will be similarly analysed with a mixed-model procedure determined by the nature of the outcome (i.e. whether binary or continuous). Full details of all statistical analyses, including the sensitivity analysis, will be described in a statistical analysis plan (SAP) which will be finalised and signed off before the master database study is locked at study completion. When finalised and signed off, the SAP will be available upon request.

### Statistical methods: handling missing data {27c}

We do not anticipate there will be a significant amount of missing data on the primary outcome given that this is extracted electronically from patient records. Patient characteristics used to stratify randomisation (site and type of presentation, i.e. self-harm or suicidal ideation), which will be included as covariates in the primary analysis, will have been collected in order to implement the randomisation. The primary analysis will include all non-missing data. This approach is valid under the assumption that outcome data are missing at random (MAR) (that is, missingness may be systematically related to covariates included in the model, but not to the missing outcome itself). If more than 5% of primary outcome data are missing, we will conduct sensitivity analyses using methods that may include multiple imputation (with an imputation model that includes predictors of missingness and auxiliary outcome measures that might be associated with the primary outcome) and pattern-mixture modelling to investigate sensitivity to departures from the MAR assumption. This will be described in further detail in the SAP described in the “Statistical methods: methods for additional analyses (e.g. subgroup analyses) {27d}” section.

### Statistical methods: methods for additional analyses (e.g. subgroup analyses) {27d}

In October 2022, the randomisation model was changed from clinician to individual patient randomisation, with the approval of the PSC. A total of 46 patients were recruited under the original study design. As of December 2024, the study achieved its target sample size.

While the components of the intervention have not changed, the timing of delivering the components has changed. In the original study design, the narrative interview and enhanced safety planning components were delivered in the ED, alongside the standard psychosocial assessment. Three solution-focused sessions were then delivered over a period of 8 weeks, and an additional bank session is available up to 9 months after randomisation. Seventeen out of 46 patients recruited under the original study design received the intervention in this form. When individual patient randomisation was implemented, all study participants received the standard psychosocial assessment in the ED, and intervention participants received their first session soon after (rather than in) the ED. All participants in the intervention arm (before and after the study redesign) received the same intervention sessions (one narrative interview and enhanced safety planning session and three solution-focused sessions plus an additional bank session). As such, all participants recruited under both designs will be included in the primary analysis.

The original inclusion criteria focused on patients presenting to the ED with self-harm. Due to increasing numbers of patients presenting with suicidal ideation, inclusion criteria were expanded to include patients with suicidal ideation. While these may be viewed as two distinct presentations, there is considerable overlap between suicidal ideation and self-harm [6, 34], and practitioners highlighted this group as needing further support. People presenting with suicidal ideation may have harmed themselves in the past and re-present to the ED with self-harm. Similarly, people presenting with self-harm may have presented with suicidal ideation in the past and re-present to the ED with self-harm [35]. Both those presenting with self-harm or suicidal ideation have been identified as warranting personalised management plans and active follow-up to reduce the risk of self-harm [6]. As such, the inclusion criteria were expanded to include people presenting with suicidal ideation, meaning the intervention is now being tested on a more heterogeneous sample. A sensitivity analysis will explore the effect of the intervention for participants recruited after the study redesign.

### Economic evaluation

The economic evaluation will follow the intention to treat principle. The aim is to assess the cost-effectiveness of the ASSURED intervention in addition to standard care in comparison to TAU. The primary analysis will be a within-trial cost-utility analysis conducted from the NHS and personal social services (PSS) perspective. The time horizon will be 18 months from randomisation. The primary outcome measure will be quality-adjusted life years (QALYs), derived using CORE-6D—a preference-based measure derived from the CORE-OM instrument [22] and patient survival data. Within-trial cost-effectiveness analysis—with the difference in the number of patients re-attending the emergency department with a referral to liaison psychiatry over the 18-month trial period as an outcome measure—will also be reported.

In sensitivity analysis, QALYs derived using EQ-5D-5L [25] instead of CORE-6D will be reported. The CORE-6D and EQ-5D-5L data will be collected at four assessment points, i.e. baseline and 3-, 9-, and 18-month post-randomisation, using the case report form.

Our cost data collection will cover cost items from the NHS and PSS perspective as well as from a wider societal perspective. The intervention cost will be collected using separate inventory forms for practitioner training and delivery of the ASSURED intervention, respectively. Participant use of inpatient, outpatient, and mental health care will be requested from NHS England. Further data on participant use of primary and community care, medications, social services, and productivity loss and family costs will be collected using the Client Service Receipt Inventory (CSRI), administered at baseline and 3-, 9-, and 18-month post-randomisation.

The economic analysis will recognise the correlation between outcomes and costs data, accounting for clusters at hospital sites and clinicians, and adjusting for key covariates (see “Statistical methods: handling missing data {27c}”). The impact of missing data on outcomes and costs data will be addressed using appropriate methods depending on the pattern and proportion of missing data. The nonparametric bootstrap method will be used to assess uncertainty around summary cost utility measures.

Sensitivity analyses will be conducted including conducting analysis from a societal perspective, using two alternative outcome measures, and evaluating the cost-effectiveness of ASSURED treatment on participants recruited after the protocol change. If the intervention demonstrates a positive effect, we will develop an exploratory analysis evaluating the likely long-term effects of the intervention using a decision analytic model. A detailed health economic analysis plan will be finalised and signed off before the study database is locked and health economists unblinded to intervention allocation.

### Process evaluation—qualitative interviews

We are aiming to conduct a concurrent qualitative process evaluation to explore the implementation and acceptability of the ASSURED intervention and understand the mechanisms of change. We are aiming to conduct interviews with 30 patients, up to 30 practitioners, up to 10 team managers and local PIs with the aim of exploring the delivery of the intervention implementation of ASSURED in the liaison setting, and approximately 10 carers or trusted others if available in the intervention arm across at least 4 sites to capture a range of local contexts. Additional interviews will be conducted with participants in the intervention group who may have experienced difficulties with drug and alcohol use, autistic people, and LGBTQI+ people to further explore their experiences in the ED and with the ASSURED intervention. We anticipate that these interviews will allow full exploration of the implementation, experiences, and context of the intervention. However, if further issues are identified during analysis that require exploration, we will interview additional participants/practitioners. The interviews will explore positive and negative experiences of the intervention.

Interviews will take place face to face or by phone/video conference, depending on the preference of participants. There is potential for these interviews to act as an intervention, so a sensitivity analysis will be carried out excluding those participants who were interviewed.

Participants for the qualitative interviews will be purposively sampled by the study team. Purposive sampling will be carried out based on data collected at post-baseline and the 3-month research interviews. We will identify people of different ages, sex, and ethnicity. The sample will include participants with positive outcomes (completed intervention and/or substantial improvements in self-harm and well-being), negative outcomes (withdrawing from the intervention and/or no reduction in self-harm and well-being), and those with a weak and strong therapeutic alliance.

Intervention practitioners will be purposively sampled to include practitioners of different sex, age, and ethnicity and from different professional backgrounds (e.g. psychiatrists, nurses, social workers, occupational therapists). If practitioners withdraw from the study, we will ask if they are willing to take part in a qualitative interview to explore their reasons for withdrawing.

Carer/trusted other participants, if available, will be purposively selected in the intervention arm to include male and female carers and, based on their relationship with the patient, to include parents, partners, and friends.

## Methods: monitoring

### Data monitoring committee: composition of the data monitoring committee and its role and reporting structure {28a}

An independent DMC was set up prior to the start of the trial and consists of one independent statistician, one experienced mental health practitioner, and one service user representative. To ensure the credibility of the decisions made by the DMC and for the integrity of the trial, all members of the DMC were invited to declare any competing financial, professional, or scientific interests with the study and the study sponsors. The DMC members have declared no competing interests. The DMC meets at least twice a year and has further email communications as necessary. Meetings are attended by the trial manager, the trial statistician (blinded), and, if necessary, the sponsor representative.

The role of the DMC is to provide advice to the trial team where necessary, advise on protocol modifications, review the accruing trial data, and monitor evidence for treatment harm. To monitor evidence for treatment harm, the DMC is presented with summaries of safety reporting data (adverse events, severity, expectedness, relatedness/causality), and members are not blinded to allocation. The trial statistician does not attend the closed report. The DMC will also assess whether there are any reasons (for example related to the safety of the intervention) why the trial should stop, although there are no formal, predefined criteria for stopping the study. It is customary that the DMC does not make decisions about the trial but rather makes recommendations to the PSC.

### Data monitoring committee: interim analyses {28b}

None.

### Trial monitoring: frequency and plans for auditing trial conduct {29}

The research will be subject to monitoring and auditing by the sponsor, delegated to the Pragmatic Clinical Trials Unit (PCTU) at Queen Mary, University of London (QMUL), as appropriate. Monthly meetings with the Trial Management Group take place. A multidisciplinary risk assessment has been conducted involving the PCTU Quality Assurance manager, CI, senior statistician, and other relevant staff members. Based on the risk assessment, the trial is considered a low-risk study and will be audited once by the PCTU. Meetings with the PSC and the PMG take place at least every 6 months to monitor adherence to the study protocol, approve changes to the study protocol, monitor study recruitment, and the overall timetable and reporting to funders.

## Ethics

### Research ethics approval {30}

London–City & East Research Ethics Committee provided ethical approval for the study on the 12th of November 2021 (reference: 21/LO/0683).

### Protocol amendments {31}

Amendments will be approved by the Research Ethics Committee (REC) and Health Research Authority (HRA). The joint sponsors and NHS Research and Development departments will be routinely informed of trial amendments.

### Consent or assent {32a}

Informed consent for study participation was received by researchers — either as part of the core study team or the local NHS research teams, with training in Good Clinical Practice and the study procedures. Under the original design of the study, informed consent was received in person in the ED prior to the psychosocial assessment.

With individual patient randomisation, potentially eligible participants were approached within a few days of having attended the ED by a member of the extended clinical team to ask if they agree to be contacted by researchers about the study. Study researchers then approached participants for informed consent remotely over the phone or video call within approximately 2 weeks from the date that the patient attended the ED.

An online information video about the study was developed as part of the initial approach, to be shown to potential participants when considering participating in the study (the video is available at https://vimeo.com/871531926/b28f23da61. A participant information sheet (PIS) was provided to participants that included relevant information about the study and their participation as well as the contact details of the study research team and local PI. The PIS was sent to participants via email or post, and participants were encouraged to take time to think about the study and discuss their participation with trusted others/carers before reaching a decision. Patients were informed that they can choose to withdraw from the study at any time without having the obligation to inform researchers about their reasons behind their decision and without their medical or legal rights being affected if they choose to do so.

In instances in which people may have experienced distress, researchers would show empathy and sensitivity, allow time, and reapproach patients at a later time when they were less distressed and better able to consider their participation in the study. For people with low literacy, in addition to sending the PIS to participants via email or post and encouraging people to discuss their participation, researchers read out the PIS and carefully took the time to answer any questions.

If patients agreed to participate in the study, a consent form was completed remotely (via telephone or videoconferencing) by the researcher. Participants were then sent the complete remote consent form to their emails or via post and had 3 days to raise any concerns or express their disagreement.

### Consent or assent: additional consent provisions for collection and use of participant data and biological specimens in ancillary studies if applicable {32b}

No additional consent provisions are required.

### Confidentiality {33}

All researchers and study staff comply with the requirements of the Data Protection Act [36] and the General Data Protection Regulation (GDPR) with regard to the collection, storage, processing, and disclosure of personal information and will uphold the core principles of both frameworks. Personal data will be stored in accordance with the Data Protection Act 2018. All participants will be assigned a participant ID number, which will be used for all data processing purposes. All hard copies of data, including sociodemographic forms, consent forms, and patient receipts, are kept in lockable filing cabinets in participating sites and are only accessible to the research team members on a need-to-know basis. Any electronic data transfer between members of the research team is carried out securely. Lists linking participant names to participant ID numbers remain with local sites and the central study team at City St. George’s, University of London. In accordance with the UK Policy Framework for Health and Social Care Research and Devon Partnership NHS Trust record management and security policies, research data will be securely archived as per Devon Partnership NHS Trust procedures and kept for 20 years. The chief investigator will be the data custodian.

### Ancillary and posttrial care {34}

Posttrial care consists of treatment as usual.

## Discussion

This study aims to explore the clinical and cost-effectiveness of a rapid, brief, and psychological intervention for adults who present to the ED after harming themselves or with thoughts of wanting to end their lives. The results of this trial are intended to improve the experiences of mental health care for both patients and practitioners and inform the implementation of the ASSURED intervention in the future. The trial will address knowledge gaps and limitations of previous studies. Currently, there are few evidence-based interventions for self-harm in the ED context in England and beyond. Those presenting to the ED with self-harm are a high-risk group for future suicide. Providing a rapid psychological intervention may offer an important opportunity to support this underserved population. The findings will contribute to the evidence base along with other current trials such as the FReSH START trial (ISRCTN73357210) [37] aiming to deliver a psychotherapeutic intervention to improve quality of life among people who repeatedly harm themselves.

The strengths of the study include strong lived experience involvement, participant recruitment from 14 EDs across a range of diverse urban and rural sites, rapid follow-up for people attending the ED with suicidality/self-harm, a long follow-up period of 18 months, and a primary outcome (ED reattendance) that can be extracted from medical records. The limitations include needing to redesign the trial because of the very challenging ED context during and after COVID-19, with 46 patients recruited before the modified trial design. However, delivering an intervention to patients soon after ED attendance may be more practical than in very busy EDs if the intervention were to be implemented in practice. Due to the impact of COVID-19 (i.e. significantly increased pressure on EDs and diverting mental health patients from EDs), there was a pragmatic decision to reduce statistical power from 90% to 80%, which may reduce the likelihood of detecting clinically meaningful effects. This would impact the primary outcome rather than secondary outcomes. There is a possibility that the completeness and accuracy of recording “referral to liaison psychiatry” in NHS digital systems may vary across different hospitals. However, we will use a predefined data extraction protocol to minimise this risk. Staff turnover has meant that more staff needed to be trained and supervised in delivering the intervention. We did not include people who required an interpreter, which limits the generalisability of the study: this would ideally be addressed in any future studies. Finally, there is a geographical imbalance in terms of the participating hospitals, with more ED sites in urban rather than rural areas of England. This may potentially affect the generalizability of findings in the future and the representativeness of the sample recruited.

Delivering a RCT in the ED context has presented many logistical challenges. However, with support from our lived experience advisory panel, funder, the research team, clinical principal investigators, and NHS R&D teams, the trial has succeeded in recruiting the target sample.

## Conclusion

ASSURED is the first large-scale multisite randomised controlled trial to test the clinical and cost-effectiveness of a rapid brief psychological intervention consisting of up to five sessions for people presenting with self-harm and/or suicidality in the UK. We hope the evidence may contribute to guiding policy and practice to improve the lives of people who present to the ED in crisis and are at increased risk of ending their lives.

## Trial status

Practitioner recruitment and training commenced in March 2022 and continued throughout the trial. Patient recruitment commenced in August 2022 and was complete in December 2024. The trial is currently in the follow-up data collection stage, which is scheduled to be complete in the summer of 2026. Currently, the trial is operating under protocol Version 10.0, dated 11.09.2025.

## Supplementary Information

Additional file 1. SPIRIT 2025 checklist of items to address in a randomized trial protocol.

## Abbreviations

AE: Adverse event
BSSI: Beck’s Scale for Suicide Ideation
CI: Chief investigator
CORE-OM: Clinical Outcomes in Routine Evaluation-Outcome Measure
CRF: Case report form
CSRI: Client Service Receipt Inventory
DMC: Data monitoring committee
ED: Emergency department
EQ-5D-5L: EuroQol-5 Dimension-5 Level tool
LEAP: Lived Experience Advisory Panel
NHS: National Health Service
NICE: National Institute for Health and Care Excellence
PCTU: Pragmatic Clinical Trials Unit
PI: Principal investigator
PIS: Participant information sheet
PMG: Programme Management Group
PSC: Programme Steering Committee
PSS: Personal social services
QALYs: Quality-adjusted life years
RCT: Randomised controlled trial
REC: Research Ethics Committee
SIX: Social Outcomes Index
SWEMWBS: Short Warwick-Edinburgh Mental Wellbeing Scales
TAU: Treatment as usual
